# Aspirin at 75 to 81 mg Daily for the Prevention of Preterm Pre-Eclampsia: Systematic Review and Meta-Analysis

**DOI:** 10.3390/jcm13041022

**Published:** 2024-02-10

**Authors:** Brielle Demuth, Ariane Pellan, Amélie Boutin, Emmanuel Bujold, Louise Ghesquière

**Affiliations:** 1 Centre de Recherche du CHU de Québec, Université Laval, Québec, QC G1V 0A6, Canada; brielle.demuth@crchudequebec.ulaval.ca (B.D.); amelie.boutin@crchudequebec.ulaval.ca (A.B.); louise.ghesquiere@chu-lille.fr (L.G.); 2 Department of Pediatry, Faculty of Medicine, Université Laval, Québec, QC G1V 0A6, Canada; 3 Department of Obstetrics and Gynecology, Faculty of Medicine, Université Laval, Québec, QC G1V 0A6, Canada; 4 Department of Obstetrics, Centre Hospitalier Universitaire de Lille, 59000 Lille, France

**Keywords:** pregnancy, pre-eclampsia, women health, aspirin

## Abstract

**Background**: Aspirin at 150 mg daily, initiated in the 1st trimester of pregnancy, prevents preterm pre-eclampsia. We aimed to estimate whether a dose of 75 to 81 mg daily can help to prevent preterm pre-eclampsia as well. **Methods**: A systematic search was conducted using multiple databases and meta-analyses of randomized controlled trials (RCTs) that compared aspirin initiated in the first trimester of pregnancy to placebo or no treatment, following the PRISMA guidelines and the Cochrane risk of bias tool. **Results**: We retrieved 11 RCTs involving 13,981 participants. Five RCTs had a low risk of bias, one at unclear risk, and fiver had a high risk of bias. A pooled analysis demonstrated that doses of 75 to 81 mg of aspirin, compared to a placebo or no treatment, was not associated with a significant reduction in preterm pre-eclampsia (8 studies; 12,391 participants; relative risk, 0.66; 95% confidence interval: 0.27 to 1.62; *p* = 0.36), but there was a significant heterogeneity across the studies (I^2^ = 61%, *p* = 0.02). **Conclusion**: It cannot be concluded that taking 75 to 81 mg of aspirin daily reduces the risk of preterm pre-eclampsia. However, given the significant heterogeneity between the studies, the true effect that such a dose of aspirin would have on pregnancy outcomes could not be properly estimated.

## 1. Introduction

Pre-eclampsia (PE) is a major cause of maternal and neonatal morbidity and affects 2% to 8% of pregnancies [1]. PE is one of the leading causes of maternal death worldwide, resulting in more than 42,000 deaths annually [2,3,4,5,6,7]. Moreover, PE can have long-term effects on both mother and newborn. In fact, it has been observed that women with PE are at increased risk of cardiovascular disease later in life, while newborns are at greater risk of delayed growth at birth, with an increased risk of obesity and cardiovascular disease later in life.

PE is now commonly subdivided into preterm PE, with delivery before 37 weeks of gestation, and term PE, with delivery at or after 37 weeks [8]. When compared to term PE, preterm PE is associated with a higher incidence of fetal growth restriction, as well as perinatal morbidity and mortality [9]. Incomplete transformation of uterine spiral arteries, diagnosed with placental bed biopsies, have been observed in 70 to 100% of preterm PE cases and only 20 to 30% of term PE cases compared to the late forms, suggesting that disorders of deep placentation are more specific to the early forms of PE [10].

Recent scientific evidence has shown that daily aspirin usage reduces the risk of preterm PE when initiated in the early stages of pregnancy [11,12]. For instance, the Combined Multi-marker Screening and Randomized Patient Treatment with Aspirin for Evidence-Based Pre-eclampsia Prevention (ASPRE) trial demonstrated that aspirin, initiated between 11 and 14 weeks of gestation and at a dose of 150 mg/day, reduces the risk of preterm PE by 62% (95% confidence interval: 26–80%) [13]. In a meta-analysis, including the ASPRE trial, Roberge et al. showed that a dose of aspirin at or above 100 mg, initiated before 16 weeks of gestation reduces the risk of preterm PE by 67% (95% confidence interval: 43–81%) [14]. Of note, they observed no significant reduction in preterm PE when aspirin was initiated after 16 weeks of gestation (relative risk of 0.59; 95% confidence interval: 0.29–1.19), and aspirin was not associated with a reduction in term PE, regardless of the dose used [14,15,16]. The latter observation supports the view that there is a pathophysiological difference between preterm and term PE.

At present, most international guidelines recommend a dose of aspirin between 75 mg and 162 mg per day in women at high risk of PE or preterm PE. The American College of Obstetricians and Gynecologists (ACOG), the Society for Maternal–Fetal Medicine (SMFM) and the US Preventive Services Task Force (UTSPSTF) recommend that high-risk pregnant women should take aspirin at 81 mg daily after 12 weeks of gestation, and for it to be continued daily until delivery [17,18]. The Society of Obstetricians and Gynecologists of Canada (SOGC) recommends a dose of aspirin between 80 and 162 mg per day, starting before 16 weeks and stopping by 36 weeks [19]. The National Institute for Health Care and Excellence (NICE) recommends a dose of 75 mg to 150 mg daily from 12 weeks until delivery [20]. The International Federation of Gynecology and Obstetrics (FIGO) published a pragmatic guide for first-trimester prediction and prevention of PE, and suggested that women weighing less than 40 kg take 100 mg of aspirin, whereas women weighing 40 kg or more should take 150 to 162 mg of aspirin per day [9].

Although many organizations support a dose of 75 mg to 81 mg daily for the prevention of PE, the evidence to confirm such an approach is limited, considering results from the ASPRE trial and meta-analysis cited above suggested that a dose of 100 to 150 mg is preferred [13,14]. Therefore, our objective was to examine the effect of 75 to 81 mg of aspirin initiated in the first trimester of pregnancy on the prevention of preterm PE.

## 2. Materials and Methods

We performed a systematic review and meta-analysis of randomized controlled trials (RCTs) that compared 75 to 81 mg of daily aspirin during pregnancy to a placebo or no treatment. RCTs were identified through a search of PubMed, EMBASE, CINAHL, Web of Science, and Cochrane Central Register of Controlled Trials (CENTRAL) from January 1985 to July 2023. The following keywords and MeSH terms related to aspirin for pre-eclampsia were used to search titles and abstracts: aspirin and/or acetylsalicylic acid, preeclampsia and/or eclampsia and/or preterm preeclampsia, pregnancy and/or pregnant women and/or women, 75 mg, 80 mg, 81 mg, low-dose, trial, and randomized trial. Reference lists and bibliographies were also searched. No language restriction was applied. The review adhered to the Preferred Reporting Items for Systematic Reviews and Meta-Analyses (PRISMA) guidelines [21] and was registered in the PROSPERO database (ID: CRD42023430816).

### 2.1. Study Selection

Included studies were RCTs that recruited pregnant women who received aspirin initiated before 16 weeks of gestation, and who were followed until delivery. The intervention was an aspirin dose between 75 and 81 mg daily, and the control was no treatment or a placebo. Studies were excluded if aspirin was associated with other treatments, if the treatment started before pregnancy, or if it was initiated after 16 weeks of gestation.

### 2.2. Data Extraction

All citations were screened independently by two reviewers. Abstracts and citations that appeared to be relevant were independently reviewed, and all potentially eligible studies (based on the inclusion criteria) were fully evaluated by the two reviewers. Disagreements were resolved by the opinion of a third party if necessary. In cases of missing data, we contacted the authors to obtain additional information.

### 2.3. Assessment of Risk of Bias

The data was extracted independently and compiled according to the article, year, numerical data, study design, and methodological quality, in duplicate. The risk of bias of each trial was assessed using the Cochrane Risk of Bias 2 tool (RoB 2) [22]. The five domains screened were: (1) bias arising from the randomization process; (2) bias due to deviations from intended interventions; (3) bias due to missing outcome data; (4) bias in measurement of the outcome; and (5) bias in selection of the reported result. Each category previously listed was independently rated as “High”, “Low”, or “Unclear” bias by two reviewers. If the two reviewers failed to agree on the risk of bias of the studies, a third reviewer was consulted to reach a consensus. The risk of publication bias was assessed by visual exploration of funnel plots and with a Egger test.

### 2.4. Data Synthesis and Statistical Analysis

Data synthesis was performed for the primary and secondary outcomes. The primary outcome was the risk of preterm PE (PE with delivery of one or more fetuses before 37 completed weeks of pregnancy). PE was defined as a systolic blood pressure ≥140 mm Hg or diastolic blood pressure ≥90 mm, Hg that occurs >20 weeks’ gestation in combination with proteinuria, defined as urinary excretion ≥300 mg protein in a 24 h urine specimen or ≥1+ protein on dipstick, or similar definitions [1]. The secondary outcome included all PE, regardless of gestational age and birth weight.

Relative risk (RR) and mean difference (MD) with a 95% confidence interval (CI) were calculated for each study and pooled for global analysis using DerSimmonian and Laird random effect to consider the variability and heterogeneity between studies. Heterogeneity was assessed with Higgins’s I^2^, and considered high if ≥50%. Analyses were carried out with the Review Manager software (version 5.3; Cochrane Collaboration, Copenhagen, Denmark).

## 3. Results

**Figure 1:**
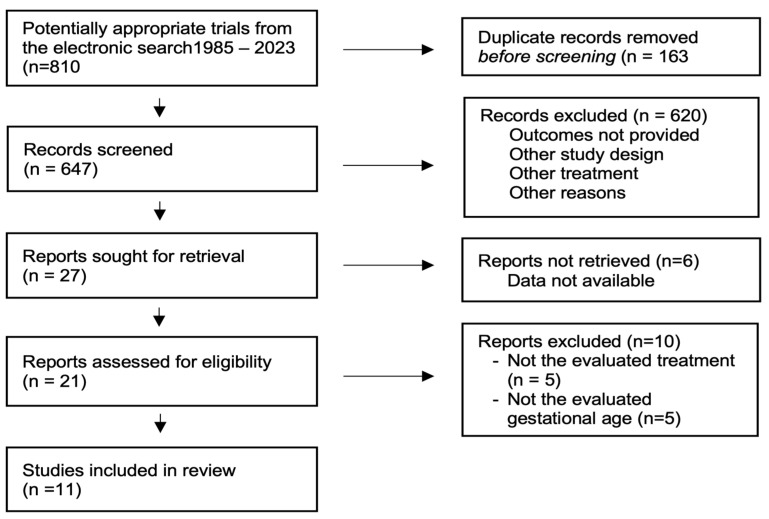
Selection of studies.

Study selection: The literature search identified 810 citations, from which 163 duplicates were withdrawn (Figure 1). After screening the titles and abstracts, 620 were excluded, and 27 articles were fully evaluated. A total of 11 RCTs, involving 13,981 participants, were used in our analysis, however, only 8 studies, involving 12,391 participants, were used for the primary outcome [23,24,25,26,27,28,29,30,31,32,33]. The characteristics of the included studies are reported in Table 1.

The RCTs were conducted in Canada, Iran, Croatia, China, USA, Barbados, Germany, India, Pakistan, Zambia, Democratic Republic of the Congo, Guatemala, and Kenya. The inclusion criteria varied from each study; however, all studies included patients at moderate or high risk of PE between 6 and 16 weeks of gestation. The sample size ranged between 30 and 11,976 participants. Incidence of preterm PE was the secondary outcome for all studies, except for one [27].

**Figure 2:**
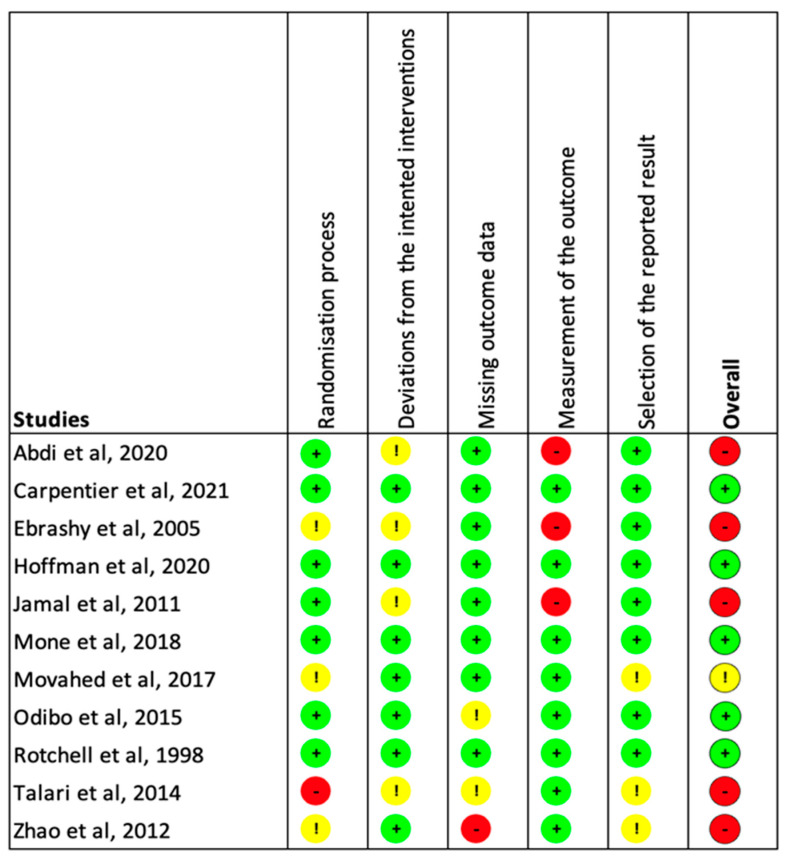
Estimation of the risk of bias among the included studies. The five domains screened were as follows: bias arising from the randomization process; bias due to deviations from intended interventions; bias due to missing outcome data; bias in measurement of the outcome; and bias in selection of the reported result. Each category previously listed was independently rated as “High” (Red), “Low” (Green), or “Unclear” (Yellow) [23,24,25,26,27,28,29,30,31,32,33].

Five studies were found to be at low risk of bias, one was found to have an unclear risk due to the randomization process and selection of the reported results, and five were found a high risk of bias due to the five domains of potential bias (Figure 2).

**Figure 3:**
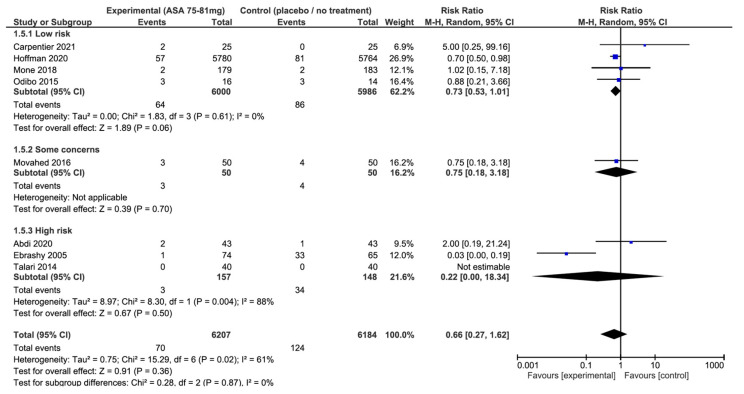
Impact of aspirin 75–81 mg vs. placebo or no treatment on preterm pre-eclampsia: forest plots stratified according to the risk of bias. We observed that aspirin at 75–81 mg daily was not associated with a lower risk of preterm pre-eclampsia when compared to placebo or no treatment, regardless the risk of bias. The relative risk for preterm pre-eclampsia among the studies with a low risk of bias is 0.73 with a 95% confidence interval of 0.53 to 1.01; *p* = 0.06.

Synthesis of results: Eight RCTs reported data for preterm PE in 12,391 participants [23,24,25,27,29,30,32,33]. The pooled analysis showed that 75 to 81 mg of aspirin taken daily was not associated with a significant reduction in the risk of preterm PE compared to placebo or no treatment groups (RR: 0.66; 95% CI: 0.27–1.62; *p* = 0.36) (Figure 3).

**Figure 4:**
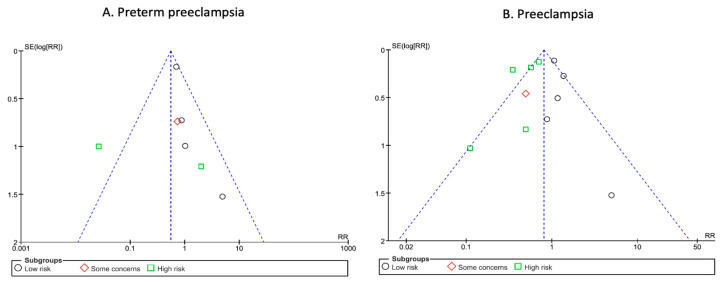
Funnel plots of included trials for preterm pre-eclampsia and pre-eclampsia. Analysis of the funnel plots suggest no publication bias for preterm pre-eclampsia (**A**) or pre-eclampsia (**B**). Egger test suggested no asymmetry of the funnel plots: preterm pre-eclampsia: *p* value 0.63; pre-eclampsia: *p* value 0.30.

In contrast, we observed a significant heterogeneity across studies (I^2^ = 61%, *p* = 0.02). When these results were stratified by risk of bias, there was also no significant difference, regardless of the risk of bias in the studies(Figure 3). Nonetheless, results from the low-risk of bias subgroup showed a near statistically significant effect and no heterogeneity (RR: 0.73; 95% CI: 0.53–1.01; *p* = 0.06; I^2^ = 0%). The funnel plots and Egger test did not suggest an asymmetry, or high risk of publication bias (Figure 4).

**Figure 5:**
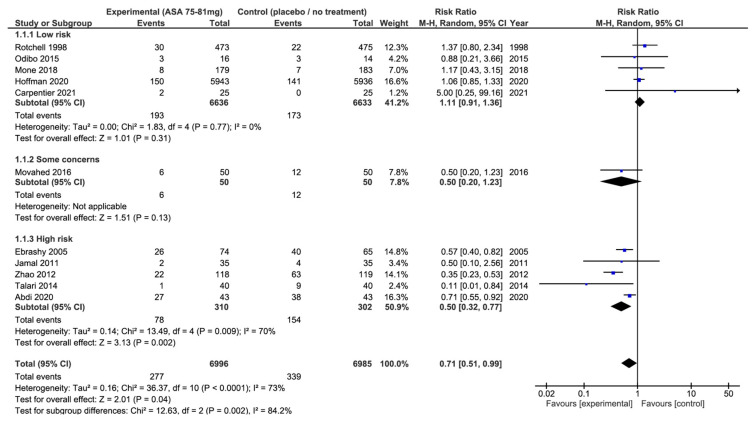
Impact of 75–81 mg aspirin vs. placebo or no treatment on pre-eclampsia: forest plots stratified according to the risk of bias. We observed that aspirin at 75–81 mg daily was associated with a lower risk of pre-eclampsia when compared to placebo or no treatment (relative risk: 0.71; 95% confidence interval: 0.51–0.99; *p* = 0.04). When these results were stratified by risk of bias, the use of aspirin was not associated with a lower risk of pre-eclampsia among the studies classified as low risk of bias, or among the studies with some concerns of the potential risk of bias when compared to placebo or no-treatment groups. On the other hand, there was a significant reduction in occurrences of pre-eclampsia among the studies classified at high risk of bias.

Regarding our secondary outcomes, we observed a significant reduction in the rate of all occurrences of PE, when aspirin intake was reduced, (11 studies, 13,981 participants, RR: 0.71; 95% CI: 0.51 to 0.99; *p* = 0.04) and a greater birth weight as well(9 studies, 4810 newborns, MD: 116 g; 95% CI: 28 to 205 g; *p* = 0.01) (Figure 5). However, there was significant heterogeneity between the studies (I^2^ = 73%, *p* < 0.01, and I^2^ = 72%, *p* < 0.01, respectively).

When these results were stratified by risk of bias, we observed that 75–81 mg of aspirin had no significant effect on the risk of all PE in the studies with a low risk of bias (5 studies, 13,269 participants, RR: 1.11; 95% CI: 0.91 to 1.36; *p* = 0.31, I^2^ = 0%, *p* = 0.77) or with some concerns (1 study, 100 participants, RR: 0.50; 95% CI: 0.20 to 1.23; *p* = 0.13), while we observed a significant reduction in all occurrences of PE in the studies with a high risk of bias (5 studies, 612 participants, RR: 0.50; 95% CI: 0.32 to 0.77; *p* = 0.04, I^2^ = 73%, *p* < 0.01) (Figure 5).

For the neonatal weight, no significant mean difference was found with 75–81 mg of aspirin versus placebo or no treatment in the studies at low risk of bias (3 studies, 4458 newborns, MD: 19 g; 95% CI: −14 to 52 g; *p* = 0.26) while significant mean differences were found in the studies with some concerns (1 study, 100 newborns, MD: 199 g; 95% CI: 2 to 397 g; *p* = 0.05) or high risk of bias (5 studies, 612 newborns, MD: 156 g; 95% CI: 29 to 282 g; *p* = 0.02) (Supplementary Materials, Figure S1).

## 4. Discussion

Our meta-analysis suggests that a daily aspirin dose of 75 to 81 mg initiated before 16 weeks of pregnancy is not associated with a significant reduction in occurrences of preterm PE. However, we observed an important heterogeneity between the studies included (I^2^ = 61%, *p* = 0.02). We observed a significant reduction in occurrences of PE, regardless of gestational age (RR = 0.71; 95% CI: 0.51–0.99, *p* = 0.04 with I^2^ = 73%, *p* < 0.01) and a greater birth weight (mean difference of 116 g; 95% CI: 28–205 g, *p* = 0.01, I^2^ = 72%, *p* < 0.01) among pregnancies exposed to 75–81 mg aspirin, but the association was not observed among studies at low risk of bias. According to these results, there is no sufficient evidence to recommend a daily dose of 75 to 81 mg of aspirin to prevent preterm PE, nor all PE, or to increase birth weights.

There are some limitations to our findings. Our conclusion is limited by the fact that there is considerable heterogeneity between the involved studies for our primary and secondary outcomes. Given the significant heterogeneity between the studies and the results stratified by risk of bias, it is difficult to estimate the true effect of 75 to 81 mg of daily aspirin on pregnancy outcomes. Second, several studies were identified to have a high risk of bias. Third, the inclusion criteria were very different between the studies. Fourth, preterm PE was the primary outcome for only one study that was considered at high risk of bias [27]. On the other hand, the key strengths of our study are that we included more than 10 randomized trials, allowing the assessment of bias using funnel plots and the Egger test; most studies used a placebo in the control group; and we included one very large multicenter trial [25]. Given the significant heterogeneity between studies and the results of studies at low risk of bias, the true effect of 75 to 81 mg of daily aspirin on pregnancy outcomes remains ambiguous.

Looking at previous literature, our findings regarding our primary outcome, preterm PE, are in line with the current available data. In fact, meta-analyses looking specifically at aspirin dosage show that a dose > 81 mg is necessary for the prevention of preterm PE. For instance, a systematic review and meta-analysis conducted by Van Doorn et al., comparing aspirin to placebo or no treatment, determined that in order to prevent preterm PE, a dose of aspirin exceeding 81 mg is necessary. Specifically, a dose of 150 mg of aspirin per day was shown to be optimal in the prevention of preterm PE after stratification based on the different doses [34]. The systematic review and meta-analysis by Roberge et al., including more than 40 studies involving 20,909 participants, using meta-regression, concluded a dose–response effect between the dose of aspirin initiated before 16 weeks of pregnancy and the reduction in preterm PE. Aspirin had no beneficial effect at 50–60 mg; however, the effect was highly significant at 100 mg daily [35]. At 50–60 mg, the beneficial effect of aspirin was absent, whereas the effect became highly significant at 100 mg daily. Finally, in a previous meta-analysis of randomized trials comparing 75–81 mg versus 150–162 mg, we found that an aspirin dosage of 150–162 mg was associated with a significant reduction in preterm PE compared to an aspirin dosage of 75–81 mg (RR = 0.34; 95% CI, 0.15–0.79; *p* = 0.01; I2 = 0%) [36]. A recent randomized trial that was not included in our meta-analysis came to the same conclusion [37]. It is also important to note that the results of the ASPRE trial were stratified according to prescription adherence, and among women who took at least half of the 150 mg aspirin tablets (equivalent to 75 mg), the reduction in preterm PE was not significant (392 participants; relative risk of 0.57; 95% confidence interval: 0.23–1.41) among those who took less than 90% of the aspirin tablets, (less than 135 mg a day) compared to a very significant reduction in preterm PE among women who took 90% of the tablets or more (1143 participants; relative risk of 0.24; 95% confidence interval: 0.09–0.65) [13].

These observations align with several studies which show that some women respond differently to similar doses of aspirin. Hence, some authors have used the term aspirin resistance, and others have used the term non-responders [38,39,40]. In fact, according to several studies, a dose of 75 to 100 mg may be insufficient to affect platelet aggregation in about one third to one half of pregnant women [23,39]. It was therefore proposed to adjust the aspirin dose according to bleeding time and/or PFA-100 using a platelet aggregation test. This approach has demonstrated a significant reduction in severe and early PE [38]. Interestingly, aspirin resistance (diagnosed using PFA-100) is much more common in patients with twin pregnancies [23]. Thus, response to aspirin may not be associated with patient weight or body mass index, as some authors have suggested [9]. These observations, in addition to the data from the current study and the literature, strongly suggest that a dose of 75 to 81 mg could probably prevent some cases of preterm and/or severe PE, but that a dose higher than 81 mg daily would be ideal for preventing the maximum number of cases among the entire population identified as being at risk.

Our study is limited by the fact that there is a lack of literature on the role of aspirin 75 mg to 80 mg in the prevention of pre-eclampsia. Indeed, there has only been one medium-sized study (over 100 randomized participants) and no large studies (over 500 randomized participants) conducted in developed countries, and none have been carried out in North America. However, the English, Canadian, and American learned societies suggest that such a dose could be used. Our systematic review was carried out to the best of our knowledge and with the tools available, however, it is possible that an important study may have been overlooked. Nonetheless, when we reviewed other systematic reviews and meta-analyses on the subject, as well as the references of clinical guidelines from many countries, we did not find any additional studies, which leads us to believe that our review was relatively complete. Furthermore, while the studies all investigated women at high risk of pre-eclampsia, we can observe that their definition was heterogeneous, as was the rate of pre-eclampsia or preterm pre-eclampsia in the no-treatment group, further suggesting that the populations studied were different. In this context, it is all the more difficult to draw any other conclusion, as the lack of adequate studies makes it impossible to recommend the 75–81 mg dose for the prevention of pre-eclampsia or preterm pre-eclampsia.

With respect to our secondary objectives, our results suggest that 75 to 81 mg of aspirin can reduce up to 30% of any PE, regardless of gestational age, and can increase the birth weight by approximately 116 g. However, there is considerable heterogeneity between studies and studies at low risk of bias do not support such effect (RR = 1.11; 95% confidence interval: 0.91–1.36). In 2007 and 2019, the Cochrane review reported that aspirin doses greater than 75 mg daily resulted in a significant reduction in the risk of PE that was superior to lower doses [41,42]. The Cochrane review did not distinguish the effect of aspirin between term and preterm PE.

Thus, when we compile all the evidence in the literature concerning the prevention of pre-eclampsia with aspirin started early in pregnancy, we find that: (1) the effect of aspirin is mainly aimed at early forms of the disease, commonly referred to as preterm PE, which is more often associated with deep placental dysfunction; (2) current data seems to suggest a possible benefit at doses of 75 to 81 mg daily—however, this is not demonstrated by high-grade scientific evidence, according to this current study; (3) doses of 100 to 150 mg are associated with a significant reduction in preterm PE and early onset PE, the most severe forms of PE; (4) recent randomized trials comparing a dose of 75–81 mg to 150–162 mg have demonstrated a superior effect with the higher doses; (5) observational studies have demonstrated an additional benefit to testing for aspirin resistance and increasing the dose in those with an inadequate response. Some authors have therefore proposed a large-scale randomized trial to compare the different doses. However, there are a few aspects to consider: (1) the negative effects of aspirin are rather rare, and that recent randomized trials have shown that it is reasonable to reduce the dosage, or even stop aspirin in the second trimester of pregnancy, avoiding potential side effects in the third trimester; (2) the positive impacts are significant for women at high risk of pre-eclampsia. Taking these factors into account, it is questionable whether it is ethically acceptable to randomize pregnant women to a dose lower than 100 mg daily without adequately informing them of the current literature and the potential benefits at the optimal dose [43,44].

## 5. Conclusions

In conclusion, the current meta-analysis suggests that an aspirin dose of 75 to 81 mg taken daily and started before 16 weeks of gestation is not effective in the prevention of preterm PE, but could have some positive effects for the prevention of PE among moderate- to high-risk women, though these remain lower than those reported with higher doses of aspirin. However, the lack of high-quality studies and, importantly, the heterogeneity of the studies used limits the clinical scope of our findings and must therefore, be interpreted in the light of other scientific evidence.

## 6. Future Directions

We must now determine which questions are the most important and urgent to answer in a context where pre-eclampsia remains a major cause of perinatal morbidity and mortality. Before conducting a randomized trial to compare aspirin doses again, there is a need to identify the issues at stake. First, it must be understood that aspirin is most effective in preventing early forms of PE, typically associated with deep placentation disorders, and rarely those at term. Recent studies have shown that aspirin significantly improves pulsatility indices assessed by uterine artery Doppler, a surrogate marker of impaired placentation [10,15,45]. Consequently, we need to be able to identify women at risk of preterm pre-eclampsia, i.e., those with profound placental dysfunction or at high risk of developing it. At present, screening for preterm PE in the first trimester using a combination of blood (placental growth factor) and ultrasound (uterine artery Doppler) biomarkers added to maternal factors (age, body mass index, and obstetrical history) appears to be the most effective method of identifying high-risk pregnant women [46]. However, once we have completed this screening, we need to inform the pregnant women that there is a dose of aspirin (150 mg) that has been shown to be effective for the prevention of preterm PE in this context (the ASPRE trial), and that a meta-analysis, our present study, of randomized trials that used a lower dose (between 75 and 80 mg) did not allow us to conclude that there was a benefit from such a dose. In this context, it is difficult to believe that a well-informed pregnant woman would be inclined to accept a randomization that could expose her to a potentially ineffective dose, all the more so considering that negative side effects are rare.

We therefore suggest the following alternatives for future major studies. First, we need to check whether it is necessary to continue aspirin beyond the second trimester of pregnancy. Indeed, considering that the effect of aspirin is probably related to an improvement in the trophoblastic invasion of the spiral arteries, which is usually completed by the end of the second trimester, it is justified to consider stopping aspirin between 24 and 28 weeks of pregnancy. The scientific arguments beyond the physiological hypotheses are multiple. Firstly, a large, randomized trial demonstrated that a dose of 150 mg aspirin per day starting at 23 weeks of gestation in pregnant women with an abnormal uterine artery Doppler had no effect on the risk of early onset PE or other pregnancy complications [47]. Secondly, a randomized trial recently demonstrated no impact on the risk of pre-eclampsia and other pregnancy outcomes from stopping aspirin at 24–28 weeks [43]. Finally, most arguments against the use of a higher dose of aspirin concern potential maternal bleeding complications that may occur during delivery, or bleeding complications in the newborn [48,49]. Thus, stopping aspirin at the end of the second trimester would reduce concerns about these potential complications, without influencing the optimal beneficial effect of aspirin during the second trimester. If a second major randomized trial confirms that it is possible and safe to stop taking aspirin at the end of the second trimester of pregnancy, without reducing its effect on the risk of preterm PE, then it will become futile to repeat a major trial comparing a daily dose of 75–81 mg to a dose of 150–162 mg.

## Figures and Tables

**Table 1:** Characteristics of included studies.

First Author (Year)	Country	N	Inclusion Criteria	Aspirin Dose	Control	GA * at Start (Weeks)	Primary Outcome
Carpentier (2022) [23]	Canada	95	Twin pregnancy	80 mg	Placebo	8 to 14	Birth weight
Abdi (2020) [24]	Iran	90	Previous history of PE	80 mg	Placebo	12 to 15	PE, IUGR, PTB *
Hoffman (2020) [25]	Several countries	11,976	Nulliparous women	81 mg	Placebo	6 to 14	PTB
Jamal (2012) [26]	Iran	70	PCOS before pregnancy, no diabetes mellitus or hypertension.	80 mg	No treatment	6 to 12	UtA * Doppler at 20 weeks
Ebrashy (2005) [27]	Croatie	139	High risk for HDP or IUGR and abnormal UtA Doppler	75 mg	No treatment	14 to 16	PE, IUGR, PTB
Zhao (2012) [28]	Chine	237	High risk for HDP or IUGR	75 mg	Placebo	13 to 16	PE, HDP
Odibo (2015) [29]	USA	30	High risk for HDP	81 mg	Placebo	11 to 14	PE
Talari (2014) [30]	Iran	80	Abnormal UtA Doppler	80 mg	Placebo	12 to 16	PE
Rotchell (1998) [31]	Barbados	948	Pregnancy before 32 weeks	75 mg	Placebo	<16	PE
Movahed (2017) [32]	Iran	100	Abnormal UtA Doppler	80 mg	Placebo	11 to 14	PE, IUGR, PTB
Mone (2018) [33]	Dublin	546	Nulliparous	75 mg	No treatment	11 to 14	Completion of research protocol

* GA: gestational age; PE: pre-eclampsia; HDP: hypertensive disorder of pregnancy; IUGR: intra uterine growth restriction; PTB: preterm birth; PCOS: polycystic ovary syndrome; FMF: fetal medicine foundation; UtA: uterine artery.

## Data Availability

No new data have been created, and all the results obtained are available in this article. Literature searches can be made available on request to the corresponding principal author.

## Supplementary Materials

The following supporting information can be downloaded at: https://www.mdpi.com/article/10.3390/jcm13041022/s1, Figure S1. Impact of aspirin 75–81 mg vs. placebo or no treatment on birth weight by risk of bias.
