# Supplementary material for: Aspirin at 75 to 81 mg Daily for the Prevention of Preterm Pre-Eclampsia: Systematic Review and Meta-Analysis
Source: J Clin Med. 2024 Feb 10;13(4):1022. doi: 10.3390/jcm13041022 (PMC10888723; doi:10.3390/jcm13041022)
Supplement: Supplementary file 1 [file jcm-13-01022-s001.zip › jcm-2831386-supplementary.pdf]

Supplementary Materials:

Figure S1. Impact of aspirin 75-81mg vs placebo or no treatment on birth weight by risk of bias.

	Experimental (ASA 75-81 mg)			Control (Placebo / no treatment)				Mean Difference
**Study or Subgroup**	**Mean**	**SD**	**Total**	**Mean**	**SD**	**Total**	**Weight**	**IV, Random, 95% CI**
**1.11.1 Low risk**								
Carpentier 2021	2,385	529	48	2,224	706	47	7.6%	161.00 [-90.27, 412.27]
Mone 2018	3,529	469	179	3,478	493	183	15.7%	51.00 [-48.11, 150.11]
Rotchell 1998	3,098	538	1819	3,086	541	1822	19.0%	12.00 [-23.05, 47.05]
**Subtotal (95% CI)**			**2046**			**2052**	**42.3%**	**18.79 [-13.97, 51.55]**
Heterogeneity: $Tau^2 = 0.00$; $Chi^2 = 1.78$, df = 2 (P = 0.41); $I^2 = 0\%$								
Test for overall effect: Z = 1.12 (P = 0.26)								
**1.11.2 Some concerns**								
Movahed 2016	3,074	384	50	2,875	600	50	9.9%	199.00 [1.55, 396.45]
**Subtotal (95% CI)**			**50**			**50**	**9.9%**	**199.00 [1.55, 396.45]**
Heterogeneity: Not applicable								
Test for overall effect: Z = 1.98 (P = 0.05)								
**1.11.3 High risk**								
Abdi 2020	2,779	755	43	2,729	722	43	5.7%	50.00 [-262.24, 362.24]
Ebrashy 2005	3,065	798	74	2,948	812	65	7.0%	117.00 [-151.37, 385.37]
Jamal 2011	3,329	537	35	3,104	359	35	9.1%	225.00 [11.00, 439.00]
Talari 2014	3,200	300	40	3,200	500	40	10.8%	0.00 [-180.70, 180.70]
Zhao 2012	2,890	340	118	2,611	479	119	15.3%	279.00 [173.31, 384.69]
**Subtotal (95% CI)**			**310**			**302**	**47.8%**	**155.57 [29.16, 281.98]**
Heterogeneity: $Tau^2 = 9983.16$; $Chi^2 = 8.13$, df = 4 (P = 0.09); $I^2 = 51\%$								
Test for overall effect: Z = 2.41 (P = 0.02)								
**Total (95% CI)**			**2406**			**2404**	**100.0%**	**116.37 [27.95, 204.79]**
Heterogeneity: $Tau^2 = 10405.25$; $Chi^2 = 28.58$, df = 8 (P = 0.0004); $I^2 = 72\%$								
Test for overall effect: Z = 2.58 (P = 0.010)								
Test for subgroup differences: $Chi^2 = 7.04$, df = 2 (P = 0.03), $I^2 = 71.6\%$								

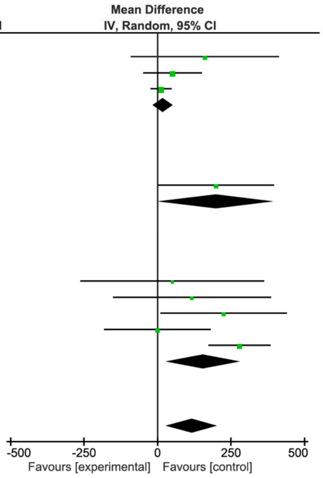

We observed that the dose of aspirin (ASA) at 75–81 mg daily was associated with a significant greater birth weight, when compared to placebo or no treatment. When these results were stratified by risk of bias, there were no significant differences for the low-risk studies and a significant difference with a greater birth weight for the moderate and high-risk studies, when compared aspirin (ASA) at 75–81 mg daily to placebo or no treatment.
